# Moderate Increase in Protein Intake Promotes a Small Additional Improvement in Functional Capacity, But Not in Muscle Strength and Lean Mass Quality, in Postmenopausal Women Following Resistance Exercise: A Randomized Clinical Trial

**DOI:** 10.3390/nu11061323

**Published:** 2019-06-13

**Authors:** Paula C. Nahas, Luana T. Rossato, Fernanda M. Martins, Aletéia P. Souza, Flávia M. S. de Branco, Marcelo A. S. Carneiro, Kely R. C. Teixeira, Fábio L. Orsatti, Erick P. de Oliveira

**Affiliations:** 1 School of Medicine, Federal University of Uberlandia (UFU), Av. Pará, nº 1720, Bloco 2U, Campus Umuarama, Uberlandia 38400-902, Minas Gerais, Brazil; nahaspaula6@gmail.com (P.C.N.); luanathrossato@hotmail.com (L.T.R.); fla-msb@hotmail.com (F.M.S.d.B.); kelraspante@hotmail.com (K.R.C.T.); 2 Exercise Biology Research Group (BioEx), Federal University of Triangulo Mineiro (UFTM), Uberaba 38061-500, Minas Gerais, Brazil; fernanda_mmartins@hotmail.com (F.M.M.); teia_depaula_souza@hotmail.com (A.P.S.); marcelo__mrcl@hotmail.com (M.A.S.C.); fabiorsatti@gmail.com (F.L.O.); 3 Department of Sport Sciences, Federal University of Triangulo Mineiro (UFTM), Uberaba 38061-500, Minas Gerais, Brazil

**Keywords:** muscle strength, dietary intervention, muscle function, muscle mass quality

## Abstract

The aim of this study was to evaluate the effect of a moderate increase in protein intake on muscle strength, functional capacity and lean mass quality improvements in postmenopausal women following resistance exercise. Forty-seven postmenopausal women were randomized in two groups: Normal protein (NP, *n* = 25), who received a dietary plan containing ~0.8 g protein·kg^−1^·d^−1^ (recommended dietary allowance—RDA recommendations); and higher protein (HP, *n* = 22), which a moderate increase in protein intake was recommended (~1.2 g protein·kg^−1^·d^−1^). Resistance training was performed for 10 weeks, three times/week. Muscle strength (handgrip strength and one repetition maximum test—1-RM), functional capacity and lean mass (LM) quality (muscle strength to lean mass ratio) were evaluated. Dietary intake was assessed by nine 24 h food recalls. After intervention, both groups increased similarly the leg extension 1-RM and handgrip strength. Regarding functional capacity tests, both groups increased the balance test score (SPPB) and 10 m walk test speed, with no differences between the groups. In addition, an increase in speed to perform the 6 min and 400 m walk tests was observed over the time, with an additional improvement in the HP group (time × group interaction; *p* = 0.007 and *p* = 0.004, respectively). About LM quality, leg extension 1-RM/leg LM improved over the time in both groups (*p* = 0.050), with no time × group interaction. All these significant changes had a low effect size. In conclusion, a moderate increase in protein intake promoted a small additional improvement in functional capacity, but it did not induce a greater increase in strength and LM quality after 10 weeks of resistance exercise in postmenopausal women. This trial was registered at ClinicalTrials.gov as NCT03024125.

## 1. Introduction

Aging promotes a progressive and generalized loss of lean mass (LM), muscle function and strength [1] mainly due to “anabolic resistance” [2,3], which can be aggravated by a combination of several factors, such as a sedentary lifestyle [4] and low protein intake [3,5]. In addition, postmenopausal period results in ovarian follicular activity loss and a reduction in estrogen production in women [6], which may promote additional effects on LM, strength and functional losses [7,8]. The reduction of muscle function can lead to difficulties in carrying out daily activities, weakness, higher risk of falls and a decrease in quality of life [9,10]. In addition to strength and functional capacity evaluation, LM quality (strength to LM ratio [11]) is another important parameter to be evaluated in this population, since it is a predictor of the risk of mobility limitation [12,13]. LM quality can be a complementary assessment to evaluate strength gains independently of muscle hypertrophy [1]. In this way, strength, functional capacity and LM quality are important parameters to be evaluated and interventions aiming to improve muscle function can increase the quality of life in older women [1,14].

Resistance exercise is a known intervention that promotes strength gains in adults [15], older adults [16] and postmenopausal women [17,18]. In addition, several studies have suggested that adequate protein intake can promote additional strength and functional capacity gains [19,20], although this is not a consensus [21,22]. A recent large-scale meta-analysis (49 studies with 1863 participants) showed that increased protein intake (by supplementation) promoted additional strength gains induced by a prolonged resistance exercise protocol in trained individuals, but not in previously untrained subjects [20]. However, the mean baseline protein intake for the individuals (both trained and untrained) was ~1.4 g/kg/day, which can justify the non-effect of increased protein intake in untrained individuals, since they probably had already reached the adequate protein intake [20]. Evaluating older adults, Finger et al. [22] performed a meta-analysis and observed that protein supplementation does not seem to promote additional strength gains; however, the studies included in the meta-analysis presented a great variation in additional protein doses (6 g to 0.8 g/kg), which makes it difficult to conclude if increased protein intake promotes further improvements in muscle function. Therefore, evaluating all these data together [20,22], it is unknown if a moderate increase in protein intake can promote additional strength and functional capacity gains when the baseline protein intake is ~0.8 g·kg^−1^·d^−1^, which is commonly ingested by untrained postmenopausal women [18,23,24].

The recommended dietary allowance (RDA) of protein intake for adults and older individuals, including postmenopausal women, is 0.8 g·kg^−1^·d^−1^ [25]. However, current evidence suggests that aging leads to “anabolic resistance” [2,3], which makes a number of expert groups to suggest an intake of a greater amount of protein (1.0–1.2 g·kg^−1^·d^−1^) for the maintenance of muscle mass and function in older adults [26,27,28]. However, it is not fully known the effects of a moderate increase of protein intake (0.8 to 1.2 g·kg^−1^·d^−1^) on muscle function. For example, Isanejad et al. (2016) evaluated a three-year follow-up cohort and showed that a protein intake higher than the RDA recommendations (~1.3 g·kg^−1^·d^−1^ vs. ~0.7–1.0 g·kg^−1^·d^−1^) was associated with better physical performance in older women [29]. However, these associations seem to be indirect, since they were no longer significant after controlling for fat mass. Tieland et al. [21] showed that a moderate increase in protein intake does not promote additional improvements in strength and functional capacity in frail older adults who followed resistance exercise protocol. However, due to the limited data in the literature, more studies are necessary to evaluate the effect of these protein recommendations. Furthermore, it is still unknown whether a moderate increase in protein intake promotes improvements in strength and functional capacity in non-frail older women.

Our research group previously showed that a moderate increase in protein intake (~1.2 g·kg^−1^·d^−1^) does not promote additional lean mass gain in non-frail postmenopausal women performing resistance exercise when compared with RDA recommendations [23]. However, since the lean mass gain does not seem to determine strength and functional capacity improvements [30,31], it is necessary to evaluate the effect of these protein recommendations on strength and functional capacity. Therefore, we aimed to evaluate the effect of a moderate increase in protein intake (~1.2 g of protein·kg^−1^·d^−1^ vs. ~0.8 g of protein·kg^−1^·d^−1^) on strength, functional capacity and LM quality improvements in postmenopausal women following a resistance exercise protocol. We hypothesized that a moderate increase in protein intake would promote additional strength, functional capacity and LM quality improvements induced by resistance exercise protocol.

## 2. Methods

### 2.1. Participants

This clinical trial was a single-blind, randomized, parallel and prospective study, conducted at the Federal University of Uberlandia and at the Federal University of Triangulo Mineiro, Minas Gerais, Brazil. Only postmenopausal women (at least one year of cessation of mensuration; self reported), who did not perform resistance exercise in the last six months, who agreed to participate and signed the written consent form were included in the study. Those who did not provide the necessary information for the development of the study, who presented orthopedic limitations, alcoholic habits, any stage of kidney disease, and/or were receiving hormone replacement were excluded. This study was approved by Federal University of Uberlandia Research Ethics Committee (protocol number 1.733.512) and Federal University of Triangulo Mineiro Research Ethics Committee (protocol number: 1.090.676); and was registered at ClinicalTrials.gov as NCT03024125.

**Figure 1:**
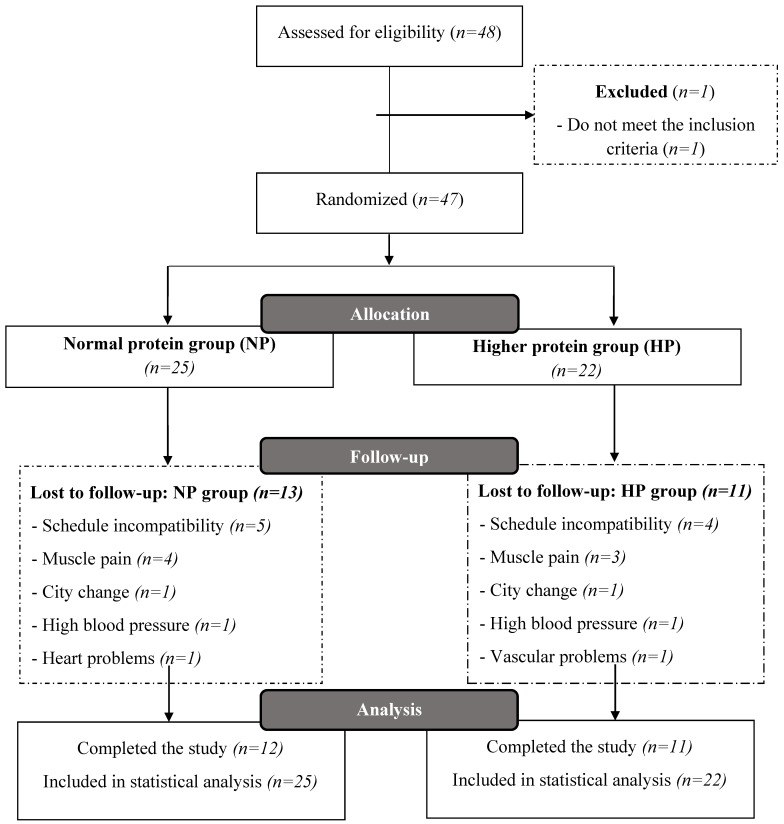
Flowchart of the individuals in the study.

In total, 48 women were initially recruited and one volunteer was excluded from the study. Once the consent was obtained, an independent researcher randomized all the participants (*n* = 47) by stratified randomization, according to the baseline values of total LM, using MedCalc^®^ software (version 11.1, MedCalc Software, Mariakerk, Belgium). After randomization, postmenopausal women were allocated in two groups: Normal protein group (NP), which received a dietary plan containing ~0.8 g protein·kg^−1^·d^−1^, as recommended by RDA; or a group that moderately increased the protein intake (higher protein intake; HP), which received a dietary plan containing ~1.2 g protein·kg^−1^·d^−1^. After the drop-out during the intervention, 12 volunteers were part of the NP group and 11 subjects in the HP group. However, all the individuals that initiated the intervention (*n* = 47) were evaluated in the present study due to the intention-to-threat analysis and statistical imputation (Figure 1).

An a priori, sample calculation was performed (G*Power v. 3.0.10, Heinrich-Heine-University Düsseldorf, Germany) for an F test (repeated measures, within-between interaction factors for two time points) to calculate the required number of participants in each group. On the basis of a statistical power (1-β err prob) of 0.80, a moderate effect size (0.25) and an overall level of significance of 0.05, 34 subjects were required for this study (17 in each group). For the power calculation (a posteriori), using a strength variable (1-RM), it was found that with 46 volunteers were necessary to have 90% of the power, making it possible to detect statistical differences. 

### 2.2. Study Design

**Figure 2:**
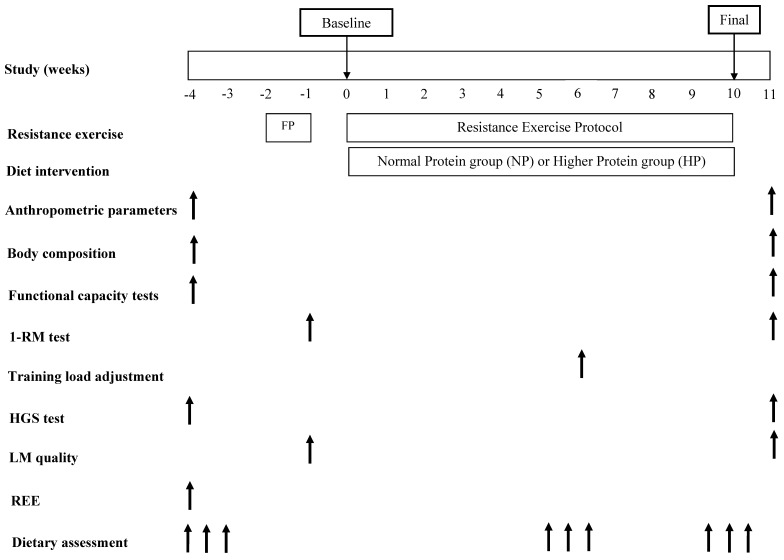
Schematic overview of the study design. Notes: *FP,* familiarization period; *1-RM,* one maximum repetition test; *HGS,* handgrip strength; *LM,* lean mass; *REE,* Resting Energy Expenditure.

Before the beginning of the study, anthropometric parameters, body composition, functional capacity tests, strength, LM quality, resting energy expenditure and dietary intake (three food recall) were assessed. These evaluations were performed for two weeks. Additionally, the volunteers also performed an adaptation training period lasting two weeks, being the first week the familiarization period and the second week was performed one maximal repetition test (1-RM). These adaptation training sessions occurred three times a week, on non-consecutive days and, after this period, the resistance exercise protocol began. At the sixth week of resistance exercise, the load was adjusted to keep the relative load. Dietary intervention and resistance exercise protocol were performed for 10 weeks. Dietary intake was assessed at the 5th and 6th weeks and at the 9th and 10th weeks, being applied six food recalls during the study. After 10 weeks intervention, anthropometric measurements, body composition, functional capacity tests, strength and LM quality were evaluated again. The study protocol is described in Figure 2.

### 2.3. Anthropometric Parameters

Weight and height were measured using a balance and stadiometer Lider^®^, respectively. Both measures were performed according to the protocol proposed by Lohman [32]. Body mass index (BMI) was calculated (body mass in kilograms divided by squared height in meters). 

### 2.4. Body Composition

Total LM, arms LM, trunk LM, arms + trunk LM, leg LM and total fat mass were assessed using dual-energy X-ray absorptiometry scanning (DXA; Lunar iDXA^®^, GE Healthcare, Madison, WI, USA) and quantified by Encore software (version 14.10, GE Medical Systems, Madison, WI, USA). The volunteers were instructed to drink two liters of water to standardize the level of muscle hydration twenty-four hours before the evaluation, and were oriented to perform eight to 10 h fasting. The volunteers wore light and comfortable clothes without the presence of metal objects. The equipment was used manually and all analyses were performed by the same researcher.

### 2.5. Functional Capacity

#### 2.5.1. Short Physical Performance Battery (SPPB)

A short physical performance battery (SPPB) [33] was composed by tests performed in the following order: Balance test, 4 m walk test and a five time sit-to-stand test. Each test score varied from 0 to 4 points and the total SPPB score varied from 0 to 12 points (sum of the scores from the three tests). For the balance test, the individuals were asked to attempt to maintain their feet in the side-by-side, semi-tandem (heel of one foot beside the big toe of the other foot), and tandem (heel of one foot directly in front of the other foot) positions for 10 s each. The time that the volunteer remained balanced was recorded. The 4 m walk test was assessed by the time walked in a distance of 4 m at a habitual gait speed. Two measures (go and come back) were recorded and the shortest time was considered as the valid measure. Lastly, the five time sit-to-stand test, postmenopausal women were instructed to fold their arms across their chests and were evaluated on the time spent in five maximum velocity squats in a chair. The technique consisted of a full sit to stand position; the volunteer started seated and the time spent was recorded.

#### 2.5.2. Six Minute Walk Test

The 6 min walk test [34] was performed in an indoor sports court. The walking course was 114 m long and it was marked every 3 m. A starting line, which marked the beginning and end of each 114 m lap, was marked on the floor using brightly colored tape. All volunteers were advised to walk as fast as possible in the 6 min of the test. The distance was recorded after the volunteer completed the test. 

#### 2.5.3. Four Hundred Meter Walk Test

The 400 m walk test [35] was performed in an indoor sports court. The volunteer was instructed to walk 400 m at a fast gait speed. A starting line, which marked the beginning and end of the course was marked on the floor using brightly colored tape. The time was recorded after the volunteer completed the test. 

#### 2.5.4. Ten Meter Walk Test

The 10 m walk test [36] was performed in an indoor sports court. The volunteer was instructed to walk 10 m at a habitual gait speed. The line marking the start and end of the route was marked on the floor using brightly colored tape. The time was recorded after the volunteer had completed the test. 

#### 2.5.5. Timed Up and Go Test

In the Timed Up and Go test [34], it was measured the time that a subject performed a stand up from a chair (without the help of hands), walked a distance of 3 m, turned around in a cone, walked back to the chair and sat down. The volunteer started sitting on a chair (with their feet on the floor and their back against the chair) and started the test after the voice command “Go”. The volunteer was instructed to walk at a fast gait speed and the time was recorded after the test was completed.

### 2.6. Strength Measurement

Muscle strength was evaluated by one repetition maximum (1-RM) and handgrip strength (HGS) tests. Before the 1-RM test, all women participated in one week familiarization (week –2 to –1: Three sessions/week, on non-consecutive days), period with low loads in order to learn the exercise techniques. On the next week (week –1), three sessions on non-consecutive days were performed to determine 1-RM. In the first session, postmenopausal women started with a warm-up at 40%–60% 1-RM and were instructed to perform eight to 10 repetitions with this load. After 1 min of rest, the load was increased to approximately 60%–80% of 1-RM, performing three to five repetitions. Again, after 3–5 min of rest, the load was considerably increased and the first attempt of the test was performed. If postmenopausal women performed only one complete movement, the 1-RM was determined. If they could not move or perform more than one repetition, a new attempt was made. After the first unsuccessful attempt, the women were rested for 3–5 min and, after this period, the load was increased or decreased. 1-RM was determined with a maximum of five attempts. If the subjects performed only one complete movement (full range of motion), the 1-RM was established and the loads used in these exercises were adopted as the maximum strength. The 1-RM test was performed for bench press and leg extension and a trained examiner performed the 1-RM measurements. The following values of the intraclass correlation coefficient (ICC; 95% confidence interval—CI) were observed in the present study: 0.93 (0.87–0.96) for bench press 1-RM and 0.97 (0.94–0.98) for leg extension 1-RM, evaluating the test-retest.

Handgrip strength (HGS) was performed with a dynamometer (Jamar^®^) adopting the unit of measure in kilograms (kg) with a scale of two kilograms. Participants were evaluated in the standing position with the arm positioned in neutral rotation and elbow flexed to 90°. The forearm and wrist were in neutral rotation. The dynamometer was positioned between the second phalange of the fingers and after the voice command of the evaluator the individuals performed the power utmost to approximate the two device rods. Three measures were taken of each hand and the highest value in each hand was considered [37]. The following values of ICC (95% CI) were observed: 0.96 (0.93–0.98) for right HGS and 0.97 (0.95–0.98) for left HGS evaluating the first and the third evaluation.

### 2.7. Lean Mass Quality

LM quality was calculated by strength to LM ratio. Bench press 1-RM and leg extension 1-RM were used as strength variables, whereas arms plus trunk LM and legs LM were used as LM variables. The ratio used in this study to determine the LM quality were bench press 1-RM/arms + trunk LM and leg extension 1-RM/leg LM.

### 2.8. Resting Energy Expenditure (REE) and Total Energy Expenditure (TEE)

The resting energy expenditure (REE) was performed by indirect calorimetry using the analyzer VO2000 (MedGraphics, Ann Arbor, MI, USA). Postmenopausal women started the test after 12 h of an overnight fast, 6–8 h of sleep, without intense physical activity in the previous 48 h of the examination, and 24 h without caffeine consumption before the test. There was a 10-min acclimatization period for reading stabilization, and then, VO_2_ and VCO_2_ were measured for 20 min [38]. Mean values of VO_2_ and VCO_2_ were used in the Weir equation for the REE measurement [39]. Additional information about the REE measurement was previously described [23]. For the calculation of total energy expenditure (TEE), the following formula was used: TEE = (REE × FA) + MET [40].

### 2.9. Dietary Assessment

Dietary intake was assessed by 24 h recalls. Three recalls were performed at baseline (4th and 3th weeks) and six during the intervention (5th and 6th/9th and 10th weeks), with a total of nine dietary recalls. Arithmetic mean was held from six recalls performed during the intervention for better representation of dietary habits. At each time of evaluation, the first 24 h recall was performed face to face and the others by phone call. In addition, 24 h recalls were performed on non-consecutive days, including two weekdays and one weekend day, at each moment of the evaluation. The assessment of dietary intake was provided by Dietpro^®^ software (version 5.7i). The United States Department of Agriculture (USDA) food composition table was used [41]. In addition, nutrition labels of manufacturers were also utilized. Total energy intake (kcal), carbohydrate (g and %), lipid (g and %) and protein (g, % and g/kg) were calculated.

### 2.10. Experimental Protocol

#### 2.10.1. Dietary Intervention

After the participants randomization into NP and HP groups, all individuals received an individualized and normocaloric dietary plan, with the list of food substitutions. All food plans were made by trained nutritionists. The NP group received a dietary plan with the protein intake proposed by RDA (~0.8 g·kg^−1^·d^−1^), while HP group received a dietary plan with a moderate increase of protein intake (~1.2 g·kg^−1^·d^−1^). In order to reach the protein recommendation for each group, high quality protein, such as meat, fish, eggs, milk and other dairy products were recommended. In addition, both groups were instructed to ingest similar amounts of protein after training (20–30 g) to standardize intake after training [42]. Dietary intervention was followed weekly by researchers using phone calls in order to know the dietary adherence and to answer possible doubts. The amount of carbohydrate prescribed was the same in both groups (~50% of total caloric value). Lipids were offered in higher amount in the NP group (25–30% of total calories) and lower in the HP group (~15% of total calories), for caloric adjustment. Postmenopausal women were blinded for the NP and HP groups. 

#### 2.10.2. Resistance Exercise Protocol

Resistance exercise was performed in a public Health and Physical Activity Center at the Federal University of Triangulo Mineiro. The training protocol followed the recommendations of the American College of Sports Medicine Guidelines for hypertrophy [43]. Resistance exercise was performed three times a week, on non-consecutive days, and at least a 48 h interval between sessions. All workouts were supervised by a qualified professional, and postmenopausal women were advised to not perform other exercise on the other days and times beyond the intervention.

The protocol consisted of dynamic exercises. Each training session started with a 10 min warm up (walking). Dynamic exercises were realized for upper and lower limbs, including a guided squat (free weight), leg curl (machine), leg extension (machine), bench press (free weight), rowing (machine), pull down (machine), triceps pulley and arm curl (free weight). The groups started with one set of each exercise in the first week and increased a set per week up to six sets for all exercises. When they reached the six series (in the sixth week), they kept this volume until the 10th week. At the sixth week of resistance exercise, the load was adjusted to ensure that postmenopausal women trained in the proposed intensity. The volunteers performed eight to 12 repetitions per set. The fixed load was 8- to 12-RMs. The interval between sets and exercises was approximately 60 s. The resistance exercise was carried out for 10 weeks, sufficient time to denote differences in muscle mass and function [44]. During the training sessions, the subjects were instructed to perform the eccentric and concentric in one second each. Exercises were supervised full time by trained professionals, who were blinded to nutritional intervention. We considered 70% as the minimum training frequency level and subjects with a lower frequency were excluded from the study.

### 2.11. Statistical Analysis

The data normality was determined using the Shapiro Wilk test. Independent *t*-test was used to compare the groups at baseline, and the values are showed as means and standard errors (mean ± SE). The multiple data imputation was performed with the insertion of plausible values for the missing data, which were imputed five times, generating five different and complete databases. The best imputation was chosen based on the values of quasi likelihood under the independence model criterion (QIC) and corrected quasi likelihood under the independence model criterion (QICC). After this, changes in dietary intake, strength, functional capacity and LM quality over the study were analyzed using generalized estimating equations (GEE) and sequential Sidak post-hoc. In addition, effect size was calculated [45], which was considered small (0.1–0.24), medium (0.25–0.36) or large (≥0.37) [46]. The reliability of strength measurements (1-RM and HGS) were tested by an intraclass correlation coefficient (ICC) and confidence interval (95% CI), which were considered as absence (0.0), poor (0.0–0.19), weak (0.20–0.39), moderate (0.30–0.59), substantial (0.60–0.79) and almost complete (≥0.80) [47]. A *p*-value of <0.05 was adopted for statistical significance. SPSS software (version 20.0, IBM Corp, New York, NY, USA) and Matlab software (MATLAB and Statistics Toolbox Release 2014a, The MathWorks, Inc., Natick, Massachusetts, United States) were used for statistical analysis.

## 3. Results

### 3.1. Baseline Characteristics

No differences were observed between groups for age, energy expenditure, anthropometric measurements, body composition, strength, functional capacity and LM quality at baseline (Table 1).

### 3.2. Dietary Intake and Training Adherence

Both groups presented similar dietary intake at baseline. Calories (kcal), carbohydrate (g and %) and lipids (g and %) intake did not change over the study in both groups. However, NP group maintained the intake of protein (g, % and g/kg), whereas the HP group increased the protein intake (g, % and g/kg) during the study (Table 2). In addition, there were no differences between the groups in relation to the training adherence.

### 3.3. Body Composition

Total lean mass (NP—pre: 36.1 ± 1.0 vs. post: 38.4 ± 1.0 kg and HP—pre: 36.6 ± 1.1 vs. post: 37.5 ± 1.1 kg) increased after the intervention (*p* < 0.001), but no differences were observed for group (*p* = 0.838) and the time × group interaction (*p* = 0.642). Arms lean mass (NP—pre: 3.9 ± 0.14 vs. post: 4.4 ± 0.14 and HP—pre: 4.0 ± 0.15 vs. post: 4.3 ± 0.15) increased after intervention (*p* < 0.001), but no differences were observed for group (*p* = 0.885) and the time × group interaction (*p* = 0.182). Arms plus trunk lean mass (NP—pre: 20.9 ± 0.60 vs. post: 21.7 ± 0.62 and HP—pre: 21.2 ± 0.57 vs. post: 22.2 ± 0.55) increased after intervention (*p* < 0.001), but no differences were observed for group (*p* = 0.607) and the time × group interaction (*p* = 0.831). Lastly, leg lean mass (NP—pre: 12.4 ± 0.48 vs. post: 12.9 ± 0.42 and HP—pre: 12.5 ± 0.51 vs. post: 12.8 ± 0.51) increased after intervention (*p* = 0.029), but no differences were observed for group (*p* = 0.990) and the time × group interaction (*p* = 0.577).

Regarding total fat mass, (NP—pre: 28.6 ± 2.2 vs. post: 28.2 ± 1.6 kg; and HP—pre: 27.6 ± 1.7 vs. post: 28.2 ± 1.6 kg) no changes were noted after intervention (*p* = 0.902), with no differences for group (*p* = 0.842) and the time × group interaction (*p* = 0.680). The effect of the intervention on body composition changes evaluating only the individuals who completed the study (without intention-to-threat analysis) can be observed in our previous publication [23].

### 3.4. Strength, Functional Capacity and Lean Mass Quality

After intervention, both groups increased leg extension 1-RM, right and left HGS, with no differences between groups. However, all these changes had a low effect size. No change in bench press 1-RM was observed in both groups over the study (Table 3).

Regarding functional capacity tests, both groups increased the balance test score (SPPB) and 10 m walk test speed, with no differences between the groups (Table 3). An increase in speed to perform the 6 min walk test and 400 m walk test was observed over the time, with additional improvements in HP group (time × group interaction; *p* = 0.007 and *p* = 0.004, respectively). In addition, both groups reduced the time to perform the 4 m walk test (SPPB), with no differences between groups. All these significant changes had a low effect size. Five time sit-to-stand test, total SPPB score, and Timed Up and Go test did not change after the intervention in both groups (Table 3). 

About LM quality, leg extension 1-RM/leg LM improved over the time in both groups (*p* = 0.050), with no time × group interaction. However, bench press 1-RM/arms + trunk LM did not change after the intervention in both groups (Table 3).

Performing the statistical analysis without multiple imputations, all the significances for group x time interaction remained the same (Table S1).

## 4. Discussion

We aimed to evaluate whether previously untrained postmenopausal women, who habitually ingested the RDA recommendations (~0.8 g of protein·kg^−1^·d^−1^), would have greater strength, functional capacity and LM quality improvements induced by resistance exercise if they moderately increased their protein intake (~1.2 g of protein·kg^−1^·d^−1^). This nutritional strategy was chosen because it allows to intake a viable amount of protein exclusively by food sources, since loss of appetite, social isolation, depression and metabolic changes can contribute to low protein intake in older adults [48]. Furthermore, the intake of ~1.2 g of protein·kg^−1^·d^−1^ is also the amount that has been proposed as a minimum quantity to be ingested by this population, since older women seem to present “anabolic resistance” [2] and is likely that a greater quantity of protein intake is needed [27,42,49,50,51,52,53]. The main finding of the present study was that a moderate increase in protein intake resulted in a small additional improvement in functional capacity, but not in strength and LM quality, in postmenopausal women following a 10 week resistance exercise protocol. 

In the present study, the resistance exercise protocol improved the functional capacity in both groups, even when shorter (4 m or 10 m walk test) or longer tests (400 m and 6 min walk test) were performed. The increase in lean mass and strength may have improved the functional capacity tests [54], which could explain the increase in walk speed in both groups. Although we observed a small effect, the moderate increase in protein intake promoted greater functional capacity improvements in longer walk tests, but not in shorter. The form the walk was performed can possibly explain these differences. In shorter tests, the individuals were instructed to perform their usual walk, whereas in longer tests they should have walked as fast as they can. Therefore, it was unlikely that a moderately increased protein intake would promote benefits in usual speed walking in non-frail postmenopausal women, which could explain the absence of effects of increased protein intake in shorter walk tests. Importantly, we showed an effect of increased protein intake in the improvement of longer walk tests independently of lean mass and strength gains, since both groups improved these parameters similarly. Thus, it is likely that increased protein intake can promote an independent effect on functional capacity improvements, but the exactly mechanism is still unknown.

There is a limited number of interventional studies comparing the effects of protein intake close to RDA recommendations with ~1.2 g of protein·kg^−1^·d^−1^ on muscle function and strength [21]. Tieland et al. compared the effect of ~1.3 g_·_kg^−1^_·_d^−1^ of protein (with protein supplementation) versus ~0.9 g_·_kg^−1^_·_d^−1^ of protein on body composition, strength and functional capacity in frail older adults who performed resistance exercise two times/week over 24 weeks. Such as in the present study, strength and physical performance improved in both groups, and no effect of increased protein intake was observed for leg extension strength, handgrip strength, SPPB and the 4 m walk test [21]. However, longer functional capacity tests were not evaluated in this study [21]; therefore, it is not possible to know if the increased protein intake had an effect in longer walk tests, as we observed in the present study. 

A possible explanation to the absence of effects of increased protein intake on strength and LM quality can be related to the training status of the individuals of the present study. A recent meta-analysis [20] showed that increased protein intake promotes additional strength gains induced by resistance exercise in trained individuals, but not in previously untrained subjects. Thus, it can partially explain the results of the present study, since we evaluated only untrained postmenopausal women. Therefore, future studies are necessary to evaluate if a moderate increase in protein intake can promote benefits on strength and LM quality in trained older women.

In the present study, LM, leg extension 1-RM, HGS, balance test—SPPB, 4 m walk test, 10 m walk test, 400 m walk test, 6 min walk test and leg extension 1-RM/leg LM were improved after the intervention. However, the other parameters evaluated did not change throughout the study. In general, this means that the exercise protocol was efficient to promote improvements in LM, strength, functional capacity and LM quality parameters in both groups. LM quality is calculated by a parameter of strength divided by LM. Thus, considering that the postmenopausal women of the present study increased leg LM, leg extension 1-RM and also leg extension 1-RM/leg LM, the exercise protocol promoted greater improvements in strength than in LM, at least in lower limbs. This could possibly be explained by the fact that LM gain does not seem to be directly associated with increases in strength [55]. However, the protein intake did not have an effect in LM quality. These improvements in strength and muscle function can have an important public health impact since they are predictors of mortality in older adults [56,57,58].

Our study had limitations. The protein intake values compared in our study were obtained through means of each group, and some individuals ingested higher or lower doses than the group average. Both groups presented underreporting of dietary intake, since the individuals related an energy intake similar to resting energy expenditure and no fat mass loss was observed. As we evaluated postmenopausal women with high body fat percentage, it was already expected since overweight and/or obese women generally underreport their energy intake [59]. This underreporting could be related to all macronutrients (including protein), which was the main dietary variable of the present study. However, the underreporting is generally related to food items rich in fat and carbohydrates, such as butter, French fries, sugars, cakes and biscuits; but not to protein food sources [60]. Therefore, it is unlikely that protein intake was underreported in our dietary data. In addition, to evaluate the LM quality we used strength to LM ratio. It is known that the use of an isokinetic strength test (strength variable) and computed tomography or magnetic resonance (muscle mass variable) can provide a more reliable evaluation of muscle mass quality. However, the form that we evaluated the LM quality is also useful and has been used by other studies [61,62,63]. At last, a large drop-out rate (~50%) occurred in our sample; however, both groups presented similar losses during the study and no differences were observed between groups at baseline when evaluated only the individuals who completed the intervention (data not shown). Nevertheless, we performed data imputation, which allowed us to evaluate all the individuals who entered in the study, maintaining the original randomization, allowing us to evaluate an intention-to-treat analysis. Even performing the imputation, we suggest that our data should be interpreted with caution due to the high drop-out rate. However, it is important to remember that this does not invalidate our data, because when we performed the analyses without statistical imputation the results remained the same (Table S1). In addition, the dietary and body composition data evaluating only the individuals who completed the study (without intention-to-treat analysis) can be observed in our previous study [23] and the results remained the same of the present study.

On the other hand, one of the strengths of the present study was the dietary intervention, which was based on increases in protein intake by various protein sources, which represents a more realistic nutritional intervention in clinical practice. Another point was the use of indirect calorimetry to measure resting energy expenditure, which is an important parameter for dietary prescription. Lately, possible errors of dietary method were minimized performing six 24 h food recalls during the intervention, in order to control the proposed dietary intervention.

## 5. Conclusions

A moderate increase in protein intake promoted a small additional improvement in functional capacity, but did not induce greater increase in strength and LM quality after 10 weeks of resistance exercise in postmenopausal women. Further studies with longer duration and/or offering higher protein amount and/or with trained individuals are needed to evaluate the effect protein intake on functional capacity and strength improvements.

## Figures and Tables

**Table 1:** Baseline characteristics of the participants.

	NP (*n* = 25)	HP (*n* = 22)	*p*-value
**Demographic and Anthropometrics**			
Age, y	62.0 ± 2.6	64.7 ±2.8	0.741
Weight, kg	67.5 ± 5.7	67.1 ± 5.0	0.908
Height, m	1.55 ± 0.02	1.55 ± 0.01	0.879
Body Mass Index, kg/m²	28.2 ± 2.0	27.5 ± 1.8	0.678
**Energy Expenditure**			
Resting Energy Expenditure, kcal	1580 ± 74.1	1431 ± 88.3	0.402
Total Energy Expenditure, kcal	2074 ± 80.4	1969 ± 79.7	0.338
**Body Composition**			
Total lean mass, kg	35.7 ± 1.1	36.5 ± 1.1	0.794
Arms lean mass, kg	4.0 ± 0.78	4.0 ± 0.81	0.883
Trunk lean mass, kg	17.3 ± 2.4	17.3 ± 2.3	0.821
Arms + trunk lean mass, kg	21.2 ± 3.1	21.3 ± 2.9	0.888
Leg lean mass, kg	12.5 ± 2.5	12.5 ± 2.6	0.847
Total fat mass, kg	28.9 ± 2.2	27.6 ± 1.7	0.931
**Strength**			
Bench press 1-RM, kg	31.0 ± 1.3	32.2 ± 1.9	0.373
Leg extension 1-RM, kg	64.2 ± 3.0	70.3 ± 4.0	0.492
Right HGS, kg	25.1 ± 1.0	26.5 ± 1.0	0.387
Left HGS, kg	23.5 ± 0.9	23.6 ± 1.2	0.800
**Functional Capacity**			
Balance test—SPPB, score	3.7 ± 0.14	3.4 ± 0.21	0.816
4 m walk test—SPPS, s	3.4 ± 0.13	3.4 ± 013	0.788
Five time sit-to-stand test—SPPB, s	11.0 ± 0.5	10.0 ± 0.6	0.167
Total SPPB, score	11.1 ± 0.2	10.9 ± 0.3	0.794
6 min walk test, m/s	1.7 ± 0.06	1.6 ± 0.04	0.322
400 m walk test, m/s	1.7 ± 0.05	1.7 ± 0.04	0.557
10 m walk test, m/s	1.3 ± 0.05	1.2 ± 0.04	0.845
Timed Up and Go test, s	7.9 ± 0.4	7.4 ± 0.3	0.294
**Lean Mass Quality**			
Bench press 1-RM/Arms + trunk LM	1.50 ± 0.05	1.51 ± 0.07	0.395
Leg extension 1-RM/Legs LM	5.35 ± 0.22	5.65 ± 0.31	0.839

Notes: Normal protein (NP), recommended dietary allowance (RDA) group; HP, higher protein group; RM, maximum repetition test; HGS, handgrip strength; SPPB, short physical performance battery; LM, lean mass. Independent *t*-test (*n* = 47). All data described in mean ± SE.

**Table 2:** Intake of calories and macronutrients according to moments and groups, with multiple imputation data.

	NP (*n* = 25)		HP (*n* = 22)		*p*-value		
	Pre	During	Pre	During	Time	Group	Time × Group
Calories, kcal	1327 ± 76.6	1375 ± 33.0	1273 ± 89.0	1463 ± 33.4	0.062	0.835	0.266
Carbohydrate, g	159.6 ± 9.4	163.0 ± 5.4	159.8 ± 12.7	170.3 ± 5.7	0.401	0.694	0.672
Carbohydrate, %	48.4 ± 1.3	48.1 ± 1.1	49.9 ± 1.0	47.9 ± 1.0	0.198	0.638	0.350
Lipids, g	53.7 ± 3.4	53.0 ± 2.0	50.7 ± 3.5	53.6 ± 1.7	0.731	0.631	0.548
Lipids, %	36.5 ± 1.2	35.2 ± 1.1	36.1 ± 0.9	33.3 ± 0.7	0.059	0.277	0.365
Protein, g	49.1 ± 2.8 ^a^	57.4 ± 1.9 ^a^	49.2 ± 2.9 ^a^	72.0 ± 3.0 ^b^	<0.001	0.029	0.012
Protein, %	14.9 ± 0.5 ^a^	17.0 ± 0.5 ^a^	15.9 ± 0.5 ^a^	19.6 ± 0.6 ^b^	<0.001	0.003	0.003
Protein, g/kg	0.76 ± 0.06 ^a^	0.85 ± 0.04 ^a^	0.76 ± 0.05 ^a^	1.17 ± 0.06 ^b^	<0.001	0.017	0.009

Notes: NP, RDA group; HP, higher protein group. Different letters represent statistical differences (*p* < 0.05). Generalized estimating equations analysis (GEE) was used to compare groups and moments with sequential Sidak post hoc (*n* = 47). All data described in mean ± SE.

**Table 3:** Strength, functional capacity and lean mass quality values according to moments and groups, with multiple imputation data.

	NP (*n* = 25)		HP (*n* = 22)		*p*-value				
	Pre	Post	Pre	Post	Time	ES (r)	Group	Time × Group	ES (r)
**Strength**									
Bench press 1-RM, kg	31.2 ± 1.2	33.5 ± 1.4	32.5 ± 1.6	32.8 ± 1.3	0.219	-	0.859	0.308	-
Leg extension 1-RM, kg	65.8 ± 2.8	72.2 ± 3.6	70.5 ± 3.5	75.1 ± 3.6	0.007	0.03	0.371	0.608	-
Right HGS, kg	25.5 ± 1.0	27.3 ± 1.0	26.6 ± 1.0	30.3 ± 0.9	<0.001	0.07	0.086	0.256	-
Left HGS, kg	23.7 ± 0.9	25.3 ± 1.0	24.0 ± 1.1	26.2 ± 0.9	0.010	0.04	0.637	0.700	-
**Functional Capacity**									
Balance test—SPPB, score	3.7 ± 0.14	4.0 ± 0.12	3.5 ± 0.20	4.0 ± 0.13	0.002	0.68	0.380	0.485	-
4 m walk test— SPPB, s	3.4 ± 0.12	3.3 ± 0.14	3.3 ± 0.13	2.9 ± 0.17	0.039	0.02	0.212	0.114	-
Five time sit-to-stand test—SPPB, s	10.9 ± 0.5	11.4 ± 0.6	9.9 ± 0.5	11.0 ± 0.7	0.106	-	0.294	0.597	-
Total SPPB, score	11.1 ± 0.2	11.1 ± 0.2	11.0 ± 0.3	11.5 ± 0.2	0.294	-	0.443	0.320	-
6 min walk test, m/s	1.7 ± 0.06	1.7 ± 0.06	1.6 ± 0.04	1.9 ± 0.06	0.002	0.04	0.778	0.007	0.07
400 m walk test, m/s	1.7 ± 0.06	1.7 ± 0.06	1.7 ± 0.04	1.9 ± 0.06	0.005	0.03	0.260	0.004	0.08
10 m walk test, m/s	1.3 ± 0.04	1.4 ± 0.04	1.3 ± 0.04	1.4 ± 0.04	0.002	0.07	0.943	0.360	-
Timed Up and Go test, s	8.0 ± 0.4	8.1 ± 0.3	7.3 ± 0.3	7.5 ± 0.3	0.520	-	0.099	0.890	-
**Lean mass quality**									
Bench press 1-RM/Arms + trunk LM	1.5 ± 0.06	1.5 ± 0.06	1.5 ± 0.06	1.4 ± 0.06	0.377	-	0.520	0.092	-
Leg extension 1-RM/Leg LM	5.4 ± 0.22	5.5 ± 0.24	5.8 ± 0.30	6.4 ± 0.36	0.050	-	0.059	0.279	-

## Supplementary Materials

The following are available online at https://www.mdpi.com/2072-6643/11/6/1323/s1, Table S1: Strength, functional capacity and lean mass quality values according to moments and groups, without multiple imputation data.
