# Supplementary material for: Moderate Increase in Protein Intake Promotes a Small Additional Improvement in Functional Capacity, But Not in Muscle Strength and Lean Mass Quality, in Postmenopausal Women Following Resistance Exercise: A Randomized Clinical Trial
Source: Nutrients. 2019 Jun 13;11(6):1323. doi: 10.3390/nu11061323 (PMC6627078; doi:10.3390/nu11061323)
Supplement: Supplementary file 1 [file nutrients-11-01323-s001.pdf]

Table S1. Strength, functional capacity and lean mass quality values according to moments and groups, without multiple imputation data.

	NP		HP		*p-value*				
	**Pre**	**Post**	**Pre**	**Post**	*Time*	*ES (r)*	*Group*	*Time x Group*	*ES (r)*
**Strength**									
Bench press 1-RM, *kg*	31.2 ± 1.2	34.2 ± 1.8	32.5 ± 1.6	33.2 ± 2.0	**0.045**	0.15	0.939	0.189	-
Leg extension 1-RM, *kg*	65.8 ± 2.8	75.0 ± 5.2	70.5 ± 3.5	77.2 ± 4.0	**0.001**	0.12	0.476	0.557	-
Right HGS, *kg*	25.5 ± 1.0	28.0 ± 1.3	26.7 ± 1.0	29.3 ± 1.3	**<0.001**	0.10	0.395	0.998	-
Left HGS, *kg*	23.7 ± 0.9	25.5 ± 1.4	23.7 ± 1.2	27.3 ± 1.3	**<0.001**	0.09	0.605	0.206	-
**Functional Capacity**									
Balance test – SPPB, *score*	3.7 ± 0.14	3.9 ± 0.08	3.5 ± 0.20	4.0 ± 0.13	**0.005**	0.13	0.692	0.160	-
4-meter walk test – SPPB, *s*	3.4 ± 0.12	3.4 ± 0.18	3.3 ± 0.13	3.0 ± 0.20	0.114	0.22	0.308	0.115	-
5-time-sit-to-stand test – SPPB, *s*	10.9 ± 0.5	11.0 ± 0.6	9.9 ± 0.5	9.9 ± 0.8	0.971	-	0.146	0.889	-
Total SPPB, *score*	11.1 ± 0.2	11.1 ± 0.2	11.0 ± 0.3	11.5 ± 0.2	0.208	-	0.594	0.332	-
6-minute walk test, *m/s*	1.7 ± 0.06	1.7 ± 0.06	1.6 ± 0.04	1.8 ± 0.04	**0.008**	0.14	0.676	**0.042**	0.15
400-meter walk test, *m/s*	1.7 ± 0.06	1.7 ± 0.06	1.7 ± 0.04	1.8 ± 0.07	0.289	-	0.653	**0.048**	0.19
10-meter walk test, *m/s*	1.3 ± 0.04	1.3 ± 0.04	1.3 ± 0.04	1.3 ± 0.04	0.272	-	0.940	0.256	-
Timed Up and Go test, *s*	8.0 ± 0.4	7.8 ± 0.5	7.3 ± 0.2	7.0 ± 0.2	0.178	-	0.087	0.639	-
**Lean mass quality**									
Bench press 1-RM / Arms+trunk LM	1.5 ± 0.06	1.5 ± 0.08	1.5 ± 0.06	1.5 ± 0.08	0.726	-	0.946	0.672	-
Leg extension 1-RM / Leg LM	5.4 ± 0.22	5.9 ± 0.32	5.8 ± 0.30	6.4 ± 0.51	**0.022**	0.13	0.300	0.700	-

Notes: *NP,* RDA group; *HP,* higher protein group; *RM,* one maximum repetition; *HGS,* handgrip strength; *SPPB,* short physical performance battery; *LM,* lean mass. Generalized Estimating Equations analysis (GEE) was used for to compare groups and moments with Sequential Sidak post hoc. All data described in mean±SE.
